# Hypocholesterolemic Activity of *Monascus* Fermented Product in the Absence of Monacolins with Partial Purification for Functional Food Applications

**DOI:** 10.1155/2014/252647

**Published:** 2014-02-20

**Authors:** Zahra Ajdari, Maaruf Abd Ghani, Mohd Khan Ayob, Saadi Bayat, Mazlin Mokhtar, Sahar Abbasiliasi, Anahita Khoramnia, Heshu Sulaiman Rahman, Parvaneh Mehrbod, Daniel Ajdari, Arbakariya B. Ariff

**Affiliations:** ^1^School of Chemical Sciences & Food Technology, Faculty of Science & Technology, MANIS Lab, Universiti Kebangsaan Malaysia, 43600 Bangi, Selangor, Malaysia; ^2^Department of Marine Biotechnology, Iranian Fisheries Research Organization, No. 297, West Fatemi Avenue, P.O. Box 14155-6116, Tehran 1411816616, Iran; ^3^Department of Chemistry, Faculty of Science, Universiti Putra Malaysia, 43400 Serdang, Selangor, Malaysia; ^4^Institute for Environment and Development (LESTARI), Universiti Kebangsaan Malaysia, 43600 Bangi, Selangor, Malaysia; ^5^Department of Bioprocess Technology, Faculty of Biotechnology and Biomolecular Science, Universiti Putra Malaysia, 43400 Serdang, Selangor, Malaysia; ^6^Faculty of Food Science and Technology, Universiti Putra Malaysia, 43400 Serdang, Selangor, Malaysia; ^7^Departmet of Microbiology and Pathology, Faculty of Veterinary Medicine, Universiti Putra Malaysia, 43400 Serdang, Selangor, Malaysia; ^8^Faculty of Veterinary Medicine, Universiti Putra Malaysia, 43400 Serdang, Selangor, Malaysia

## Abstract

Hypercholesterolemia is one of the most common chronic diseases in human. Along with chemical therapy traditional medication is used as hypocholesterolemic remedy, however, with unfavorable side effects. Recently, *Monascus* fermented product (MFP) has become a popular hypocholesterolemic natural supplement. In the present study, the hypocholesterolemic activity of *Monascus purpureus* FTC5391 fermented product ethanolic extract (MFPe) was investigated in hypercholesterolemic rats. Results showed that MFPe not only reduced the serum total cholesterol (TC), LDL-C, TG concentration, and TC/HDL-C ratio but also increased the HDL-C. Further, solid phase extraction (SPE) was carried out to obtain the hypocholesterolemic bioactive fraction. The high polar fraction of SPE increased the HDL-C (42%) and decreased the TC (53.3%), LDL-C (47%), and TG (50.7%) levels as well as TC/HDL-C ratio (69.1%) in serum. The GC-MS results of the active fraction revealed two main compounds, isosorbide and erythritol, which act as coronary vasodilator compounds.

## 1. Introduction

Today, the importance of serum lipid profile regulation particularly low-density lipoprotein cholesterol (LDL-C) concentration to prevent coronary disease has been clearly proven [1, 2]. Diet, exercise, and medication have been considered to regulate the serum lipid profile.

Several types of hypocholesterolemic medicines are available in the market, which can be taken alone or in combination with other medications. Due to unpleasant side effects of these medications, there has been considerable increase in investigation for complementary or alternative therapies, which are marketed as “natural” treatments. Hence, nutraceuticals and functional foods have attracted much interest as possible alternative therapies for lowering plasma total cholesterol (TC). Specifically, it has been most applicable for hypercholesterolemia patients, whose blood cholesterol level is marginally high (5.2–6.2 mmol/L) but not too high to warrant the prescription of cholesterol-lowering medications [3].

*Monascus* fermented product (MFP), unique Chinese traditional fermented cooked rice produced by *Monascus *spp., is one of the best representatives of nutraceuticals, which is advertised as a therapy for hyperlipidemia. The genus *Monascus* is a nonpathogenic filamentous fungus belonging to the *Ascomycetes *class and the *Monascaceae* family.

Nearly two-thirds of antihyperlipidemic activity of MFP is associated with monacolins as natural statins [4]. Monacolins inhibit 3-hydroxy-3-methyl-glutaryl coenzyme A (HMG-CoA) reductase as converter of HMG-CoA to mevalonate in initial stage of cholesterol biosynthesis pathway. Moreover, *Monascus* can produce a wide range of metabolites with additive or synergistic effects of monacolins on serum lipid profile [5–7]. On the other hand, *Monascus *spp. are fungi with extraordinary ability to produce a broad range of secondary metabolites mostly useful in human life. Hence, it is believed that MFP consumption as natural product has the potential of multifunctional therapies [5, 7, 8]. Therefore, it might be the reason that MFP consumption increased by nearly 80% from 2005 to 2008 in the United States, with sales of $20 million in 2008 [9, 10]. These days, different commercial brands of MFP are found in the market as hypocholesterolemic natural products. Several studies have shown no monacolin existence in some commercially available dietary MFP supplements in the market [11–13]. To the best of our knowledge, there were no reports in the literature regarding the clinical or *in vivo* investigation of the MFP hypocholesterolemic activity when no monacolin is included. Our previous study demonstrated the deficiency of monacolin production by *Monascus purpureus* FTC5391, a local isolate [14]. However, hypocholesterolemic activity in oral administration in rats was observed. Thus, this study was aimed at determining ethanolic extract of *Monascus purpureus* FTC5391 (MFPe) for hypolipidemic activity in experimental animals. Further, the partial purification of MFPe was carried out by solid-phase extraction (SPE) method and hypolipidemic activity of each fraction was assessed on the serum lipid profile of hypercholesterolemic rats. The hypocholesterolemic active fraction was suggested for functional food applications.

## 2. Materials and Methods 

### 2.1. Materials

Yeast extract, malt extract, casamino acid, agar, and peptone were purchased from Difco Laboratories, Inc. (Detroit, MI, USA). Other chemicals were purchased from Merck KGaA (Darmstadt, Germany). Mevinolin (MK, lovastatin) as standard and, analytical grade trifluoroacetic acid (TFA) were purchased from Sigma-Aldrich, Inc. (St. Louis, MO, USA). The red yeast rice (RYR) capsules were obtained from Cosway M Sdn Bhd (Kuala Lumpur, Malaysia).

### 2.2. Microorganism

The fungus *M. purpureus *FTC5391, isolated from local sources, was obtained from the Culture Collection Unit at the Malaysian Agricultural Research and Development Institute (MARDI). The culture was maintained on potato dextrose agar slants at 4°C and subcultured monthly.

### 2.3. Fermentation

Submerged fermentation was carried out in 1000 mL Erlenmeyer flasks containing 500 mL of fermentation medium. The medium was composed of g/L, sucrose, 100; yeast extract, 2.3; casamino acid, 5; NaNO_3_, 1.2; KH_2_PO_4_, 1; KNO_3_ 1.6; MgSO_4_·7H_2_O, 0.5; KCl, 1.6; FeSO_4_, 0.01; potato starch, 1.3; and dextrose, 17.3 [15, 16]. Fermentation was initiated by a 10% fungal culture inoculation (v/v) followed by incubation in a rotary orbital shaker at 30°C with 150 rpm agitation for 25 days. Growth cell kinetics was evaluated in the process duration. Sampling (2 mL) was carried out every 48 h interval. After that, the sample was filtered through a preweighed filter paper, and then the filter paper included biomass was dried in an oven at 90°C for 24 h, until a constant dry weight was attained.

### 2.4. Sample Preparation

**Figure 1:**
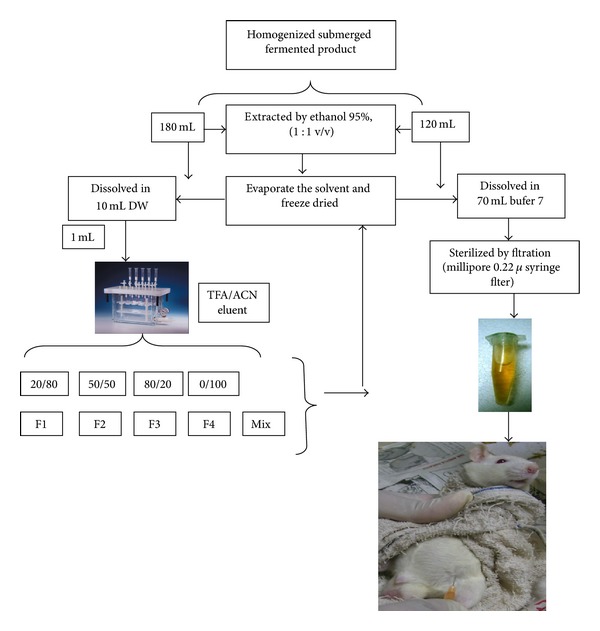
The pattern of sample preparation. F = fraction.

The flow of sample preparation in this study is summarized in Figure 1.

#### 2.4.1. Extraction

Following 25 days of fermentation, the fermented product was homogenized using a high-pressure homogenizer (EmulsiFlex-C50 CSA10, Avestin, Canada) at 1000 bar, 5 cycles, and 4°C. Afterwards, the homogenized sample was subjected to ethanolic extract with 95% ethanol (1 : 1 v/v), incubated overnight and subsequently repeated for three times in agitation condition for 2 hr at 60°C, and then centrifuged at 3000 ×g for 10 min.

#### 2.4.2. Fractionation

Solid phase extraction (SPE) system was employed for the partial purification with the following expatiation: Supelco Visiprep DL (disposable liner) SPE Vacuum Manifold, 12-port model with a GAST Diaphragm Vacuum Pump, Capacity: 1.1 open flow UI^+^ Vacuum: 24′′ HG, Motor Spec: 1/8 HP (230/50/1PH). The column was SPE-R30230B-06S, C18 (17%), 6 mL, 1 g SiliCycle. The fractionation was carried out with 10 mL of the dried crude ethanolic extract dissolved in 1 mL distilled water and applied in the column. The column was conditioned with 6 mL of ACN, followed by 6 mL distilled water. Stepwise elution was performed with 6 mL of each of the following solvents for three times: trifluoroacetic acid (TFA) 0.1%/acetonitrile (ACN) 80/20, 50/50, 20/80 and acetonitrile (100%). The fractions were analyzed by Ultra Performance LC (ACQUITY UPLC Waters Inc.) and dried under pressure.

### 2.5. Equipment and Analytical Procedure

#### 2.5.1. Ultra Performance Liquid Chromatography (UPLC)

The ACQUITY UPLC (ultra performance liquid chromatography) separation system (Waters Inc., Milford, MA, USA) was employed throughout this study. This system was equipped with an ACQUITY UPLC Sample Organizer, Column Manager and Heater/Cooler, Binary Solvent Manager, and Sample Manager, for customized throughput and condition requirements. The raw data were detected by a photo-diode array detector and acquired and processed by Waters Millennium32/Empower software, chromatographic workstation loaded on an IBM computer. To consider resolution, running time, and solvent-saving, the column of ACQUITY UPLC BEH C18 column 2.1 × 100 mm, 1.7 *μ*m was used. The chromatography was performed using a linear gradient of ACN (eluent A) and 0.1% TFA (eluent B) with a flow rate of 1 mL/min. Eluent A was increased from 35 to 75% in 9 min and kept at 75% for 4 min and then reduced to 35% in 1 min. The photo-diode array (PDA) detector was used in the wavelength ranging from 210 to 350 nm. The column temperature was set at 30°C and the injection volume was 10 *μ*L.

#### 2.5.2. Gas Chromatography-Mass Spectroscopy (GC-MS)

GC-MS analysis was conducted using a GC-MS QP5050A Shimadzu instrument fitted with an electron spray ionization source at the Department of Chemistry, Faculty of Science, Universiti Putra Malaysia. The following method and conditions were set for experiments: 0.50 *μ*L of sample injected into ZEBRON ZB-FFAP 30 m × 0.25 mm I.D *χ* 0.25 *μ*m film thickness column and helium (1 mL/min) was used as a mobile phase. Sampling time was 1 min, injection initial temperature 230°C, interface temperature 250°C, and running time was 40 min. Mass spectra were recorded under electron impact ionization at 70 eV energy. Shimadzu GC-MS solution software was used for the data acquisition. The mass spectrum was compared to a mass spectral reference library of National Institute Standard and Technology (NIST) 08S which has more than 62,000 patterns.

### 2.6. Animal Studies

#### 2.6.1. High-Cholesterol Diet Preparation

High-cholesterol-supplemented feed (2% cholesterol and 0.2% choleic acid) was prepared by first dissolving 200 g of solid cholesterol in 1 L of chloroform and then 20 g of solid choleic acid in 200 mL of glacial acetic acid, separately, with the latter mixture held in a 60°C water bath to complete dissolution. The solutions were mixed and then spread onto 10 kg of normal chow and the solvent was allowed to evaporate.

#### 2.6.2. Sample Preparation and Dosage

Based on the pervious study of animal (Sprague-Dawley rats) oral dosage (10 mL/kg bw/day of submerged fermented product) (unpublished data), 120 mL of homogenized submerged fermented sample was extracted by ethanol 95%, (1 : 1 v/v). The solvent was evaporated at 50°C afterwards freeze-dried. The dried sample was dissolved in 70 mL sodium phosphate buffer (pH 7, 50 mM). Samples were sterilized by filtration (Millipore 0.22 *μ* syringe filter) and divided into 1 mL tube according to body weight of each rat then injected to rat's peritoneal section (5 mL/kg body weight), two times injection per day for 7 days with six replications.

For partial purification, 180 mL of homogenized submerged fermented sample was extracted by ethanol 95%, (1 : 1 v/v). Each 10 mL of ethanolic extract sample was dried and dissolved in 1 mL distilled water then applied to SPE C18 column to take a fraction. In the end, four fractions were collected and one more fraction achieved by mixing these fractions. The solvent of samples was evaporated at 50°C and dried by lyophilization. Each fraction was dissolved in 70 mL sodium phosphate buffer (pH 7, 50 mM). Further, the samples were sterilized by filtration (Millipore 0.22 *μ*m syringe filter) and divided into 1 mL tube according to body weight of each rat then injected to rat's peritoneal section (5 mL/kg body weight), two times injection per day for 7 days with six replications (Figure 1).

Two positive controls were used: RYR commercial MFP and atorvastatin as the most common hypocholesterolemic medication available in the market. In the case of RYR commercial MFP, based on the previous study (unpublished data), 20 g of RYR commercial MFP was extracted with a fresh ethanol/water solution (75 : 25). The solvent was evaporated at 50°C and then the dried sample was dissolved in 70 mL sodium phosphate buffer (pH 7, 50 mM). Samples were sterilized by filtration (Millipore 0.22 *μ* syringe filter) and then injected to rat's peritoneal section, (5 mL/kg body weight) two times injections per day for 7 days with six replications. For atorvastatin, each 20 mg tablet was dissolved in 10 mL sodium phosphate buffer (pH 7, 50 mM) and injected to rats (25 mg/kg body weight) two times injection per day for 7 days with six replications.

#### 2.6.3. Animal Grouping

Twenty-four male Sprague-Dawley rats of 4 weeks of age and weighing 120 ± 10 grams were obtained in the first step (ethanolic extraction of MFPe) and forty-two rats for the second step (SPE fractionation assay). The animals were housed at two rats/cage and were allowed to feed on commercial food and water *ad libitum* throughout the study. In the first step, the rats were randomly divided into four groups (*n* = 6) comprising: a positive control (high cholesterol, HChol) and three peritoneal injection groups individually treated with RYR (HChol-C), MFPe extract HChol-E, and atorvastatin (HChol-D).

In the second step, the rats were randomly divided into 7 groups (*n* = 6), comprising: a positive control (high cholesterol, HChol), with the following injection treatment groups: TFA/ACN 80/20 (HChol-F1), TFA/ACN 50/50 (HChol-F2), TFA/ACN 20/80 (HChol-F3), ACN 100% (HChol-F4) mixture of all fractions (HChol-M), and atorvastatin (HChol-D).

All rats were fed with cholesterol-enriched diet (normal diet + 2% cholesterol and 0.2% choleic acid) for two weeks. Blood samples were collected 3 times: initially (before starting a high-cholesterol diet), second time (after one week rest and feeding with high-cholesterol diet before the treatment), and third time (one week after treatment). Serum lipid profiles including total cholesterol (TC), triglyceride (TG), high-density lipoprotein cholesterol (HDL-C) and low-density lipoprotein cholesterol (LDL-C) concentrations, and alanine transaminase (ALT) and aspartate aminotransferase (AST) activities (two enzymes representing liver-enzyme damage index) were assayed using commercial enzymatic kits.

Food was removed 24 hr before sacrificing. Animals were anesthetized and killed by diethyl ether inhalation. Liver samples were collected to evaluate the liver injury. One part of the liver was carefully removed and rinsed frequently with 0.8% sodium chloride solution for eliminating any blood and subsequently immersed in 10% formalin stock. The liver tissue was examined for damage under microscope for liver biopsy. The experiment was reviewed and approved by the Animal Care and Research Ethics Committee of the Universiti Putra Malaysia.

### 2.7. Statistical Analysis

One-way ANOVA (analysis of variance) and LSD (least square differences) in Post-hoc tests (*P*-value < 0.05) were used for data analysis. The differences in mean values were expressed as mean ± SD. Student's *t* test was used to compare the mean values of two groups; analysis of variance linear correlations was used to analyze the variety of means of multiple groups and linear correlation of different parameters using PASW statistics software (Version 18).

## 3. Results 

### 3.1. Culture Growth

**Figure 2:**
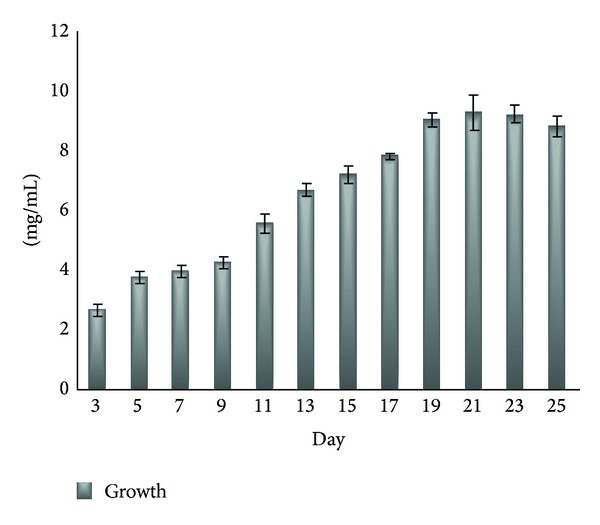
Growth kinetics for *Monascus purpureus* FTC5391 during fermentation.

Figure 2 shows the growth kinetics for *Monascus purpureus* FTC5391 during fermentation. There was an increase in cell mass production by 19th day of fermentation (exponential phase) and then a constant growth rate by 23rd day (stationary phase), afterwards there was a reduction by 25th day (declining phase).

### 3.2. Metabolite Assessments

Analysis of the MFPe by UPLC-PDA with scanning wavelength between 210 and 350 nm and GC-MS results detected almost 100 metabolites in this product.

#### 3.2.1. UPLC-PDA Analysis

**Figure 3:**
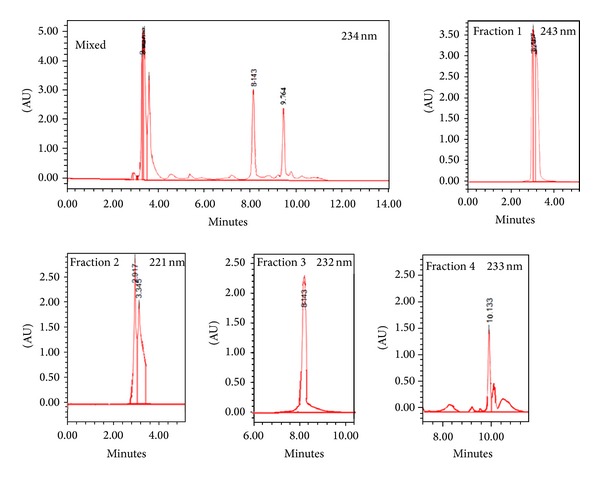
Chromatogram of SPE ethanolic extract fractions of *Monascus purpureus* submerged fermented product analyzed by UPLC-PDA. Fraction 1, Fraction 2, Fraction 3, and Fraction 4, elute with TFA 0.1%, acetonitrile/TFA 0.1% 50/50, acetonitrile/TFA 0.1% 75/25, and ACN 100%, respectively (mobile phase: acetonitrile/TFA 0.1, injection volume 10 *μ*L, flow rate: 1.0 mL/min).

Stepwise elution with a gradient of TFA/ACN [TFA 0.1% (fraction 1), TFA/ACN (50/50) (fraction 2), TFA/ACN (25/75) (fraction 3)], and ACN 100% (fraction 4) solvents using SPE yielded a bioactive hypocholesterolemic treatment on hypercholesterolemic rats in TFA 0.1% fraction.  Figure 3 shows the chromatograms of SPE fractions injected in UPLC/PDA. The chromatograms of the fractions were detected at wavelengths of 234, 243, 221, 232, and 233 nm for mixed, and fractions 1 to 4, respectively, in which most metabolites were numbered and their relative quantities noted. The UV spectra were obtained by scanning the absorbance from 210 to 350 nm.

#### 3.2.2. GC-MS Analysis

The chromatogram of MFPe and active fraction from GC-MS indicated 95 and 44 compounds, respectively. The mass spectrum of the compounds was compared with the spectrum of known compounds existed in the NIST library. The most similar spectrum of known compound name, molecular weight, similarity, formula, structure of the components of the MFPe, and bioactive fraction was clarified. Tables 1 and 2 show summarized metabolites identified by GC-MS in MFPe and bioactive fraction, respectively. In the case of MFPe mainly 17 prominent compounds comprised 78.63% of the total. Major contained compounds were 2-Furancarboxaldehyde 22.05% > Propanoic acid propyl ester 12.91% >, 4H-Pyran-4-one, 2, 3-dihydro-3, 5-dihydroxy-6-methyl 8.02% >, 1-Propanol 2, 2-dimethyl 7.03%, altogether 50.01%. On the other hand, the dominant compounds in the bioactive hypocholesterolemic fraction included 7 with 87.17% of the total. Glycerin 44.24% >, Isosorbide 11.33% >, 1,2,3,4 Butanetetrol 16.57% were the major compounds in the bioactive fraction, respectively, and they were 75.14% altogether.

### 3.3. Simple Method to Collect the Bioactive Fraction

Regarding the animal study, which demonstrated hydrophilic fraction with high hypocholesterolemic activity, a simple method was conducted to extract and the collection of the active fraction. The ethanolic extract was dried at 60°C and then washed with distilled water for 2 hr at 50°C. The analysis of this fraction showed the same result of the first fraction of SPE method. This method is a simple economical way to reach a bioactive fraction in order to obtain partial purification.

### 3.4. Animal Studies

Results of animal studies indicated that the body weight and daily intake of rats increased normally and did not show significant differences among the various groups during the experiments. In addition, the externals and health conditions of all the experimental animals showed normal expression.

The results of serum lipid profiles and ALT and AST concentrations in rats were analyzed and summarized in Tables 3 and 4. These data clearly showed a significant reduction (*P* < 0.05) in serum HDL-C level, while an increase in serum concentrations of TC, LDL-C, TC/HDL-C, ALT, and AST as well as the ratio of TC/HDL-C (atherosclerotic index) among all the experimental groups in comparison with the baseline in the second serum collection.

On the other hand, after one week of treatment, serum lipid profiles, ALT and AST concentrations in rats showed significant differences between all tested serum indicator concentrations in the second and third serum collections (*P* < 0.05).

#### 3.4.1. Evaluation of Serum TC and LDL-C Levels

Tables 3 and 4 display no significant differences among the experimental groups for the serum TC and LDL-C levels after one week of feeding by high cholesterol-diet (*P* > 0.05). The serum TC level was 4.7–7.9 mmol/L where it was 3.3–6.5 mmol/L higher than the baseline. As compared to base line, the serum LDL-C level was increased around 3.31–6 mmol/L in groups fed with high cholesterol diet. The results of the one-way ANOVA test demonstrated the significant differences for serum TC and LDL-C levels among the experimental groups after one week of peritoneal injection treatment (*P* < 0.05).

After one week of hypercholesterolemic rats' peritoneal injection treatment, serum TC level was reduced significantly (*P* < 0.05) by 52%, 44%, and 31% on the HChol-D, HChol-E, and HChol-C groups, respectively, as compared to before treatment. LDL-C reduced by 15% in the HChol group which was not a significant reduction (*P* > 0.05) but significantly decreased by 48%, 47%, and 28% on the HChol-D, HChol-E, and HChol-C groups, respectively, as compared to before treatment (*P* < 0.05).

The comparison between serum TC and LDL-C levels pre and posttreatment of MFPe fractions groups displayed reduction of 10% for TC and 8.5% for LDL-C in Hchol, 59.7% for TC and 62.7% for LDL-C in Hchol-F1, 29.6% for TC and 32.2% for LDL-C in Hchol-F2, 10% for TC and 15.6% for LDL-C in Hchol-F3, 0% for TC and 5.2% for LDL-C in Hchol-F4, 32.8% for TC and 29.8% for LDL-C in Hchol-M, and 53.3% for TC and 47% for LDL-C in Hchol-D. These results revealed that the maximum and minimum reduction of serum TC and LDL-C levels were happening in Hchol-F1 and Hchol-F4 groups, respectively. Nevertheless, differences between the serum TC level of the Hchol-F1, Hchol-D, and Hchol-M groups before and after treatments were significant (*P* < 0.05), but for other groups were not significant. However, differences between the serum LDL-C level before and after treatment were significant (*P* < 0.05) in the Hchol-F1 and Hchol-D groups.

#### 3.4.2. Evaluation of Serum TG Levels

TGs are one of the major groups of fats in the blood, which are mainly obtained from the diet. These are stored in different body tissues and used as a source of energy. The combination of high TG, high LDL-C, and low HDL-C levels increases the risk of cardiovascular diseases [17].

Compared with the baseline, serum TG concentrations were slightly higher in the experimental groups on a high-cholesterol diet. These differences noted 0.06–0.43 and 0.15–0.91 mmol/L in the MFPe and fractions of MFPe treatment groups, respectively. However, after one week of treatment, serum TG concentrations showed significant decrease in the HChol, HChol-E, and HChol-D groups at 31.5%, 42.7%, 35.86%, respectively, and 26.6% increase in the HChol-C (*P* < 0.05).

The comparison of serum TG levels pre and posttreatment displayed a significant decrease of 47.8%, 69.5%, 55.8%, and 50.7% in Hchol, Hchol-F1, Hchol-M, and Hchol-D groups, respectively (Table 4). While, there was no significant decrease in Hchol-F2, Hchol-F3, and Hchol-F4 groups with 38.5%, 42%, and 45% decrements, respectively.

#### 3.4.3. Evaluation of Serum HDL-C Levels

Rats carry the majority of cholesterol in HDL particles, which is different from human [18]. The concentration of serum HDL-C, representing “good” cholesterol, plays an important role in regulating blood cholesterol level. In this study, HDL-C concentrations were significantly less in the rat groups fed the high-cholesterol diet than at baseline (*P* < 0.05). These results demonstrated that, in rats fed with a high-cholesterol diet, serum TC and LDL-C concentrations raised, whereas serum HDL-C concentrations decreased.

The concentration of HDL-C (0.41 and 0.45 mmol/L) was marked as a baseline (Tables 3 and 4). Feeding rats with a high-cholesterol diet caused a significant decrease in HDL-cholesterol level (0.17-0.18).

The comparison of serum HDL-C levels of pre and posttreated animals demonstrated a significant increase of 47% and 32% in HChol-D and HChol-E groups, respectively, while there was no serum HDL-C level changing in HChol-C and HChol groups (Table 3). On the other hand, serum HDL-C variations in the treatment by MFPe fractions in comparison with pre and posttreatment displayed a significant increase of 63.6%, 63.2%, 40%, and 42% in Hchol-F1, Hchol-F4, Hchol-M, and Hchol-D, respectively, and no significant increase of 10.5% in Hchol-F2. While, there was no significant difference between second and third serum collection in serum HDL-C levels in Hchol-F3 and Hchol groups.

#### 3.4.4. Evaluation of Serum TC : HDL-C Ratio

The ratio of TC to HDL-C is a factor noted for evaluating the efficiency of hypolipidemic agents. The TC/HDL-C ratio at baseline was 3.8–4. This ratio was found to increase between 24–43 (Table 3) and 27−37 (Table 4) units among the high-cholesterol-fed groups, that showed hypercholesterolemia. Analysis of the third serum collected showed significant decrease of 65% and 55% on the HChol-D and HChol-E groups, respectively, but there was no significant decrease on the HChol-C group (20%) (*P* > 0.05) (Tables 3 and 4).

The comparison between atherogenic-indices of pre and posttreatment displayed significant reductions of 74.5%, 50.6%, and 69.1% in Hchol-F1, Hchol-M, and Hchol-D groups, respectively, and no significant reduction in Hchol-F2, Hchol-F3, and Hchol-F4 (34.4%, 11% and 33%), respectively (Tables 3 and 4).

#### 3.4.5. Evaluation of Serum AST and ALT Levels

Different commercial products contain high levels of citrinin, which is a type of mycotoxin causing liver and kidney damage [19]. There are some enzymes in the blood, which have been known as liver damage indicator such as ALT and AST. Generally the amounts of serum AST and ALT are considered as the most important tests to detect liver injury, where ALT is more specific than AST [20]. Thus, they can be used as markers to assess the extent of liver damage.

The toxicity or the potential of the treatments to cause liver damage was evaluated by measuring ALT and AST liver enzymes in the sera of all treated groups.

The serum AST levels of rats fed by hypercholesterolemic diet were significantly increased (*P* < 0.05), while ALT did not show any significant increase. Compared to before treatment only the serum AST levels of HChol-E reduced significantly (*P* < 0.05). The assessment of liver biopsy did not show any changes by all groups (Tables 3 and 4).

### 3.5. Detection of Monacolins and Citrinin

Some strains of *Monascus* could produce citrinin which is a mycotoxin that causes liver and kidney damage and might contaminate its fermented product [21]. The chromatography finger-printing profile demonstrated neither monacolins nor citrinin was detected in MFPe supplements. The chromatogram and UV spectrum of red yeast rice revealed 4 peaks with a similar spectrum of monacolin K. The amount of monacolin K in red yeast rice was 0.5 mg/g.

## 4. Discussion 

There is no doubt that fermentation is the most ancient subject in biotechnology, which has been a valuable source for driving beneficial compounds in modern biotechnology. Fermented *Monascus* products have been used as food and health remedies for over 1000 years in China, from which lots of wonderful agents with a broad range of applications were extracted. However, the *Monascus* potential to produce a secondary metabolite is still an interesting subject for research.

The most MFP prestige is due to important isolated secondary metabolites as a lipid profile regulator, monacolins, particularly monacolin K. So far close to 14 monacolins from MFP have been reported [5, 22, 23].

Although the majority of antihyperlipidemic activities of MFP seem to be attributed to the monacolins [4], few studies in human [24] and animal [6] found that the effect of MFP on lipid profile could not be restricted to its monacolin alone, instead, it might be the effect of combined monacolins and other substances in MFP. Ma et al. [6] demonstrated that MFP that contained 8.8 mg/g monacolin K had greatly enhanced fecal acidic sterol extraction by 3-4 folds. It was a significant step to determine the observed hypocholesterolemic activity of MFP is not only from monacolins but also due to other substances in MFP that increased bile acid extraction.

In our pervious study *M. purpureus* FTC5391 fermented product (MFPe) cultivated by modifying an inducer sporulation medium [16, 25] showed strong hypocholesterolemic activity in the absence of monacolins as compared to other MFP cultivated by different media that were administered into the rats (unpublished data). In this regard, MFPe was selected for further hypocholesterolemic study. The ethanolic extract of MFPe that demonstrated a great effect on hypercholesterolemic rats compared to atorvastatin and RYR was injected to the HChol-D group and HChol-C group to establish treatment for positive control groups. Subsequently MFPe was fractioned for partial purification and applied for hypocholesterolemic activity on rats.

High-cholesterol diet feeding has been regularly the easiest way to increase serum cholesterol levels to obtain hypercholesterolemic animals. Since, in the case of rats, excessive cholesterol in the body is immediately converted to bile acids and removed in bile fluid, a cholesterol diet for rats must contain cholesterol and bile acids such as choleic acid. The results of the present study showed that the supplementation of a cholesterol diet (2% cholesterol and 0.2% choleic acid) administered into the rats has had a significant increase in the serum TC, LDL-C, TG levels, and TC/HDL-C ratio and decrease in HDL-C compared to the baseline (*P* < 0.05).

However, variation in serum TC concentrations in the HChol group from the second serum collection to the third collection showed downregulation. A healthy body always tries to maintain the body's internal environment in good balance and does so by upregulation or downregulation. The conclusion from this observation was that, when viewing the degree of a decrease in a lipid profile, it must be considered that the decrease was caused by both the treatment and a downregulation.

The pattern of treatment effect on hypercholesterolemic rats after one week of treatment was HChol-F1 > HChol-D > HChol-E > HChol-M > HChol-C. Although, these data indicated that the serum TC, LDL-C, and TG levels were significantly decreased among all groups (*P* < 0.05), serum TG level and TC/HDL-C ratio were not significantly reduced in the HChol-C group. Serum HDL-C level significantly increased on all indicated groups (*P* < 0.05) where there was no significant change in HChol-C group. TC, LDL-C, HDL-C, TG, and TC/HDL-C are all risk factors for atherosclerosis. It is no doubt that the regulation of all these factors at the same time would be a good chance to keep the body in healthy condition and prevent atherosclerosis and cardiovascular disease.

All researchers in this area believed that the statins and RYR could regulate serum TC and LDL-C [26–28]. However, the significant effect of statins and RYRs on serum HDL-C, TG levels and TC/HDL-C ratio regulation is debatable [8, 23, 28]. Since the level of serum LDL-C is related to TC, the variation of LDL-C could be associated with downregulation in LDL receptors by the TC differences in the serum.

This study demonstrated that RYR, a commercial fermented product, has significantly reduced the serum TC and LDL-C (*P* < 0.05). There are several reasons involved for the event: in one hand, the effect of statin on lipid profile is dose-dependent and varies from one person to another, on the other hand, the effect of fermented *Monascus* product is dependent on the strain, fermentation condition, and dosage as well as the synergic or antagonistic effects of other compounds that exist in RYR besides monacolins as natural product.

The current study indicated for the first time that MFPe has remarkably regulated lipid profile in the absence of monacolins. Since MFPs' cholesterol-lowering activity is believed to be mainly attributed to its active ingredient, monacolins, it seems other bioactive hypocholesterolemic compounds in MFPs have been under monacolins shadow. Moreover, polar fraction of MFPe displayed more activity than MFPe. Indeed, fractionation process might eliminate some compounds with antagonist effect. Hence, this fraction could be a proper fraction to apply as hypocholesterolemic supplement.

GC-MS analysis of MFPe identified 95 compounds where 17 of them made up the 78% of the total. Most of these compounds were shown to have antibacterial activity. Although we could not find any report of hypocholesterolemic activity of major compounds, 9,12-Octadecadienoic acid or linoleic acid (2.47%) as an essential fatty acid and 9-octadecenoic acid (oleic acid) (2.98%) have been reported to have hypocholesterolemic activity by decreasing of LDL-C and increasing HDL-C [29].

The most active fraction of MFPe was a highly polar compounds fraction. This fraction showed reddish color, in which the existence of red soluble pigment compound was expected. GC-MS analysis of this fraction identified 44 compounds where 7 of them made up the 87.17% of the total. Two major compounds in this list include isosorbide with 95% similarity and 11.33% of total and 1, 2, 3, 4-Butanetetrol (Erythritol) with 96% similarity and 16.57% of the total. Isosorbide mononitrate is a drug used principally in treating angina pectoris which acts by dilating the blood vessels to reduce the blood pressure. It has also been used broadly as a cervical ripener to reduce delivery time at hospital [30]. Erythritol, a four-carbon sugar, found in algae, fungi, and lichens is twice as sweet as sucrose and is used as a coronary vasodilator [31]. These two compounds might be effective on lipid profile regulation. However, further studies need to be conducted on this subject.

The existence of citrinin as a mycotoxin causing liver and kidney damage has been reported in some of MFP [13]. Serum ALT and AST levels were assayed to evaluate whether or not the MFPe causes liver damage in the rats. The hepatoprotection activity of MFP against chemically induced liver injuries of rats had been reported by Aniya et al. [32]. As shown in Tables 3 and 4, serum AST level of rats was significantly increased after feeding by hypercholesterolemic diets, while serum ALT level was not significantly increased in comparison with baseline. Massive amounts of cholesterol supplement may cause liver injury and simultaneously elevate the hepatic fibrosis [33]. Nevertheless, no changes in the liver biopsy was observed, this might be due to short time of experimental period. However, by looking at hypercholesterolemic rats treated in different groups only in Hchol-E a significant reduction in serum AST (*P* < 0.05) was noticed. This reduction in Hchol-E turned to be close baseline. This observation might have been due to the existence of a bioactive MFPe compound with a moderation effect in severe liver injury, which probably has been missed in Fraction 1. Therefore, from this study it was achieved that the absence of monacolins in the ethanolic extract of *Monascus purpureus* FTC5391 fermented product was not only safe but also made an effective product to regulate lipid profile of rats as animal model.

Present evidence showed short-term beneficial effects of MFPe and its high polar fraction on lipid modification in the absence of monacolins. On the other hand, the extraction method of hypocholesterolemic active fraction of MFPe to prepare an efficient hypocholesterolemic supplement was simple and cost-effective. However, further studies are necessary to identify the substances responsible for this activity with their synergistic and antagonist effects as well as underlying molecular mechanisms, long-term effects, and safety.

## 5. Conclusion 

In conclusion, the results of the current study clearly showed that ethanolic extract of *Monascus purpureus* FTC5391 fermented product in the absence of monacolins has reduced not only serum TC and LDL-C levels but also serum TG level and TC/HDL-C ratio. Moreover, it increased serum HDL-C levels and regulated serum AST levels as liver damage enzyme. On the other hand, the high polar fraction of MFPe showed a great hypocholesterolemic activity. Isosorbitol and erythritol with 95% similarity identified by GC-MS were included in active fraction. These two compounds were reported as coronary vasodilators. However, further study is needed to identify these compounds and confirm their hypocholesterolemic activity properly. Cost-effective partial purification method in this study provides possibility to use high bioactive fraction as supplement alone or food additive.

## Figures and Tables

**Table 1:** The main compounds identified in MFPe by GC-MS.

No.	R. time	% Total	*m*/*z*	% SI	Molecular weight	Compound	Application
1	9.212	3.68	60.05	100	C_2_H_4_O_2_	Acetic acid	Preservative, making cellulose acetate
2	9.477	1.73	95.1	83	C_5_H_8_N_2_	8-3-1H Imidazole	Imidazole derivatives have occupied a unique place in the field of medicinal chemistry
3	9.55	2	95.1	92	C_5_H_4_O_2_	Furfural	An important renewable, nonpetroleum based, chemical feedstock
4	10.281	12.91	57.1	87	C_6_H_12_O_2_	Propanoic acid propyl ester	Preservative in feed and food industry
5	10.447	1.24	101.10	80	C_11_H_20_O_2_	1 3-dioxolane 2-cyclohexyl-4 5-dimethyl	Use of a steroid for enhancement of skin permeability
6	10.742	7.30	57.10	87	C_5_H_12_O	1-Propanol 2,2-dimethyl	
7	11.845	1.94	98.10	94	C_5_H_6_O_2_	2-furanmethanol	Used for resins production
8	14.633	3.77	91.10	97	C_8_H_10_O	Phenylethyl Alcohol	Antimicrobial activity
9	16.123	1.3	60.05	97	C_8_H_16_O_2_	Octanoic acid (caprylic acid)	Antibacterial in food industry
10	18.154	8.02	144.10	88	C_6_H_8_O_4_	4H-Pyran-4-one,2,3-dihydro-3,5-dihydroxy-6-methyl	DNA strand-breaking substance
11	18.914	2.18	56.10	80	C_4_H_10_N_2_O	2-Propanamine, N-methyl-N-nitroso	A precursor to many herbicides
12	20.295	22.05	97.10	92	C_6_H_6_O_3_	2-Furancarboxaldehyde	Flavoring agents
13	20.646	1.33	67.10	78	C_19_H_34_O_2_	9,12 octadecadienoic acid methyl ester	Essential oil
14	20.758	1.78	91.10	78	C_13_H_18_O_2_	Acetic acid, phenyl-isopentyl ester/Benzeneacetic acid 3-methylbutyl ester	Flavoring agents
15	23.458	2.42	60.10	94	C_16_H_32_O_2_	Hexadecanoic acid (palmitic acid)	Mainly used to produce soaps, cosmetics, and release agents
16	25.792	2.98	55.10	91	C_18_H_34_O_2_	9-Octadecenoic acid (oleic acid)	Emulsifying agent
17	26.487	2.47	67.10	92	C_18_H_32_O_2_	9,12-Octadecadienoic acid	Essential oil and industrially is used in making quick drying oils

Percentage of main compounds in whole extract		78.63					

R. time: retention time; SI: similarity.

**Table 2:** The main compounds identified in water fraction of MFPe by GC-MS.

No.	R. time	% Total	*m*/*z*	% SI	Molecular weight	Compound	Application
1	8.726	2.26	60	95	C_2_H_4_O_2_	Acetic acid	Preservative, making cellulose acetate
2	21.798	3.79	146	84	C_7_H_14_O_3_	Propanoic acid, 2-hydroxy-, 2-methylpropyl ester	
3	21.950	4.44	104	86	C_4_H_8_O_3_	3,4-Furandiol, tetrahydro-, trans	Antimicrobial
4	22.365	11.33	146	95	C_6_H_10_O_4_	Isosorbide	Angina pectoris treatment
5	22.860	1.54	144	86	C_6_H_8_O_4_	4H-Pyran-4-One, 2,3-Dihydro-3,5-Dihydroxy-6-Methyl	Aroma compound in a dairy product
6	23.917	47.24	92	95	C_3_H_8_O_3_	Glycerin	Moisturizer
7	44.602	16.57	122	96	C_4_H_10_O_4_	1,2,3,4-Butanetetrol	Coronary vasodilator

Percentage of main compounds in whole extract		87.17					

R. time: retention time; SI: similarity.

**Table 3:** Evaluation of hypocholesterolemic effect by ethanolic extract of *M. purpureus* FTC5391 fermented broth cultivated using modified inducer sporulation medium to experimental rats.

Group	TC mmol/L	TG mmol/L	HDL-C mmol/L	LDL-C mmol/L	TC/HDL-C	AST mmol/L	ALT mmol/L
Basal parameters	1.4 ± 0.13	0.67 ± 0.23	0.45 ± 0.14	0.69 ± 0.12	3.8 ± 0.63	210.7 ± 12.2	45 ± 8
HChol							
Pretreatment	4.7 ± 1.7^a^	0.73 ± 0.11^a^	0.18 ± 0.024^a^	4 ± 1.4	26.4 ± 7.7^a^	282.7 ± 43.7	54 ± 14.4
Posttreatment	4.5 ± 1.7	0.5 ± 0.1^a∗^	0.18 ± 0.04^a^	3.4 ± 1.2	25 ± 8.5^b^	323 ± 51.8^b^	67.2 ± 11.4
HChol-E							
Pretreatment	6.9 ± 1.2^ b^	1.1 ± 0.33^b^	0.22 ± 0.03^b^	6 ± 2.5	32 ± 5.7^a^	270 ± 44.3	50 ± 17.6
Posttreatment	3.9 ± 0.72*	0.63 ± 0.12^a∗^	0.29 ± 0.03^b∗^	3.2 ± 0.6*	14.3 ± 3.6^a∗^	216.7 ± 18^a∗^	51.2 ± 11.4
HChol-D							
Pretreatment	7.9 ± 2.2^b^	0.92 ± 0.36^ab^	0.17 ± 0.03^a^	6.4 ± 3	47.7 ± 18^b^	239 ± 21	51 ± 5.8
Posttreatment	3.8 ± 1.2*	0.59 ± 0.23^ a∗^	0.25 ± 0.06^ab∗^	3.3 ± 1.2*	16.6 ± 7^ab∗^	265.8 ± 47^ab^	54.5 ± 15.8
HChol-C							
Pretreatment	6.8 ± 1.7^b^	1.1 ± 0.38^ab^	0.25 ± 0.05^ab^	6 ± 2.5	28.6 ± 9.3^a^	235 ± 50.7	50.5 ± 15.6
Posttreatment	4.7 ± 0.92*	1.5 ± 0.33^b^	0.25 ± 0.08^ab^	4.3 ± 0.6*	23 ± 8.7^b^	283.5 ± 45^b^	56.2 ± 16

Hchol: high-cholesterol diet; HChol-E: high-cholesterol diet treatment by ethanolic extract submerged medium fermentation [sucrose (100 g/L), yeast extract (2.3 g/L), casamino acid (5 g/L), NaNO_3_ (1.2 g/L), KNO_3_ (1.6 g/L), KH_2_PO_4_ (1 g/L), MgSO_4_·7H_2_O (0.5 g/L), KCl (0.5 g/L), FeSO_4_ (0.01 g/L), potato starch (1.3 g/L), dextrose (17.3 g/L)]; Hchol-D: treatment by Atorvastatin and Hchol-C: red yeast rice commercial supplement treatment, data are presented as means ± SD (*N* = 6). Mean values within each column with different superscripts are significantly different (*P* < 0.05); *: shows significant different in column at (*P* < 0.05) as compared to before treatment. TC: total cholesterol; TG: triglyceride; AST: aspartate aminotransferase; ALT: alanine transaminase; HDL-C: high-density lipoprotein cholesterol; LDL-C: low-density lipoprotein cholesterol.

**Table 4:** Evaluation of hypocholesterolemic effect with *M. purpureus* FTC5391 submerged fermented (different SPE fraction) experimental rats performance serum TC, TG, LDL-C, HDL-C, ALT, and AST levels.

Group	TC mmol/L	TG mmol/L	HDL-C mmol/L	LDL-C mmol/L	TC/HDL-C	AST mmol/L	ALT mmol/L
Basal parameters	1.4 ± 0.2	0.76 ± 0.22	0.41 ± 0.16	0.63 ± 0.22	4.1 ± 1.4	208.7 ± 37.8	48.2 ± 8.9
HChol							
Pretreatment	7 ± 2.7	2.3 ± 1.1^c^	0.2 ± 0.045	5.9 ± 2.3	34 ± 11.1	308 ± 48^b^	78 ± 14
Posttreatment	6.3 ± 2.5^cb^	1.2 ± 0.53^b∗^	0.2 ± 0.05^a^	5.4 ± 2.4^b^	32.3 ± 16.5^c^	328 ± 30^ab^	82 ± 26
HChol-F1							
Pretreatment	7.7 ± 2.3	1.9 ± 0.89^bc^	0.22 ± 0.04	6.7 ± 2	35.27 ± 11.8	307 ± 42^ab^	76 ± 12
Posttreatment	3.1 ± 0.25^a∗^	0.58 ± 0.22^a∗^	0.36 ± 0.08^b∗^	2.5 ± 0.25^a∗^	9 ± 2.1^a∗^	306 ± 68^ab^	72 ± 10
HChol-F2							
Pretreatment	7.1 ± 2.8	1.3 ± 0.63^b^	0.19 ± 0.02^b^	6.3 ± 2.6	36.6 ± 14.8	295 ± 60^b^	75 ± 15
Posttreatment	5 ± 1^b^	0.8 ± 0.25^ a^	0.21 ± 0.04^a^	4.4 ± 1^b^	24 ± 6.3^bc^	300 ± 31^b^	71 ± 12
HChol-F3							
Pretreatment	7 ± 2.2	0.95 ± 0. 43^a^	0.19 ± 0.07	6.4 ± 2	41.4 ± 22.7	291 ± 63^b^	67 ± 9
Posttreatment	6.3 ± 1.5^c^	0.55 ± 0.1^ a^	0.19 ± 0.09^a^	5.4 ± 1.2^b^	36.8 ± 13.9^c^	308 ± 60^ab^	65 ± 16
HChol-F4							
Pretreatment	6.5 ± 1.2	0.91 ± 0.37^a^	0.19 ± 0.05	5.8 ± 1	37.5 ± 13.2	274 ± 35^b^	65 ± 10
Posttreatment	6.5 ± 1^c^	0.5 ± 0.1^a^	0.31 ± 0.1^b∗^	5.5 ± 1.2^b^	25 ± 12.2^bc^	287 ± 26^a^	66 ± 11
HChol-M							
Pretreatment	6.7 ± 0.65	1.47 ± 0.58^b^	0.2 ± 0.04	5.7 ± 0.7	35 ± 10.1	292 ± 46^b^	63 ± 12
Posttreatment	4.5 ± 1.5^ab∗^	0.65 ± 0.2^ a∗^	0.28 ± 0.06^ab∗^	4 ± 1.5^ab^	17.3 ± 7.5^b∗^	295 ± 40^a^	64 ± 13
HChol-D							
Pretreatment	6 ± 1.3	1.5 ± 0.52^b^	0.21 ± 0.06	5.1 ± 1.5	31.4 ± 16.8	369 ± 78.7^a^	78 ± 14
Posttreatment	2.8 ± 0.7^a∗^	0.74 ± 0.27^a∗^	0.3 ± 0.05^b∗^	2.71 ± 0.6^a∗^	9.7 ± 2.9^a∗^	355 ± 57^b^	82 ± 26

Basal parameters: all rats fed on normal diets before starting Hchol diet; Hchol: high-cholesterol diet; HChol-F1, F2, F3, and F4 high-cholesterol diets: treatment with different SPE fractions (TFA 100%, TFA/ACN 50/50, TFA/ACN 25/75, and THF 100%) of *M. Purpureus* submerged fermented [sucrose (100 g/L), yeast extract (2.3 g/L), casamino acid (5 g/L), NaNO_3_ (1.2 g/L), KNO_3_ (1.6 g/L), KH_2_PO_4_ (1 g/L), MgSO_4_·7H_2_O (0.5 g/L), KCl (0.5 g/L), FeSO_4_ (0.01 g/L), potato starch (1.3 g/L), dextrose (17.3 g/L)], and Hchol-D: treatment with atorvastatin (50 mg/kg bw/day), data are presented as means ± SD (*N* = 6). Mean values within each column with different superscripts are significantly different (*P* < 0.05); *: shows significant different in column at (*P* < 0.05) as compared to before treatment. TC: total cholesterol; TG: triglyceride; AST: aspartate aminotransferase; ALT: alanine transaminase; HDL-C: high-density lipoprotein cholesterol; LDL-C: low-density lipoprotein cholesterol.
